# Phlebotomine Fauna (Diptera: Psychodidae) and Putative Vectors of Leishmaniases in Impacted Area by Hydroelectric Plant, State of Tocantins, Brazil

**DOI:** 10.1371/journal.pone.0027721

**Published:** 2011-12-07

**Authors:** Maurício Luiz Vilela, Carina Graser Azevedo, Bruno M. Carvalho, Elizabeth F. Rangel

**Affiliations:** 1 Laboratório de Transmissores de Leishmanioses, Instituto Oswaldo Cruz/FIOCRUZ, Rio de Janeiro, Rio de Janeiro, Brasil; 2 Secretaria da Saúde do Estado do Tocantins, Núcleo de Leishmanioses, Coordenadoria de Doenças Vetoriais e Zoonoses, Palmas, Tocantins, Brasil; University of Georgia, United States of America

## Abstract

**Background:**

Although leishmaniases are regarded as serious public health issues in the State of Tocantins, as consequence of the impact of environmental changes, small advances in taxonomic and ecological studies of Phlebotominae fauna are taking place in this state. The present study aimed to improve the knowledge about the sand flies, as well as about the aspects of the bioecology of leishmaniases vectors from Porto Nacional, a city that was directly impacted by the construction of Luís Eduardo Magalhães Hydroelectric Plant (HEP – Lajeado).

**Methodology/Principal Findings:**

Sand flies were collected monthly using CDC light traps and Shannon traps for a period of 40 consecutive months, at different monitoring stations, where 7162 specimens were collected and 48 species were detected. Among the species found, 22 are first records in the state and seven are considered important vectors of leishmaniases. *Lutzomyia longipalpis*, the vector of American Visceral Leishmaniasis (AVL) showed higher frequency in urban compared to rural areas, and *Nyssomyia whitmani*, the vector of American Cutaneous Leishmaniasis (ACL), predominated in rural areas. The frequency and habits of sand fly vectors are discussed considering environmental characteristics and climatic factors.

**Conclusions/Significance:**

The construction of dams requires a great amount of labor, therefore attracting people from elsewhere. Increased migration, without adequate structure, leads to bad living conditions in new and unplanned settlements. It also leads to deforestation associated with environmental impacts, which can facilitate the spread of leishmaniases.

This study discusses the importance of *Lu. longipalpis* and *Ny. whitmani* on the transmission cycles of leishmaniases in Porto Nacional and the record of *Bi. flaviscutellata* in periurban area of the city.

## Introduction

Currently, leishmaniases are serious public health issues in Brazil. American cutaneous leishmaniasis (ACL) has been recorded in all states and is undergoing territorial expansion, revealing changes in its epidemiological profile [1]. American visceral leishmaniasis (AVL) is urbanized and has spread to the metropolitan areas of large and medium-size cities, including state capitals, occurring in all geographical regions of the country [2]. The transmission dynamics of *Leishmania spp.* are associated with a variety of complex eco-epidemiological scenarios, which offer appropriate conditions for interactions among parasite, vector and reservoirs.

In the last ten years, the State of Tocantins has recorded high rates of ACL and AVL, where 137 among 139 municipalities had already recorded human cases of ACL, and a considerable number of cities presented human cases of AVL. Porto Nacional is a city located in the area of direct influence of HEP Lajeado. The construction of this hydroeletric plant had significant environmental impacts after dam building, mainly related to deep changes in vegetation and to the building of new houses, without prior planning.

In fact, not only in Brazil, but also in other american countries the environmental impacts of human action may provide new epidemiological profiles of ACL. This context may also contribute to the urbanization of the diseases, since transmission cycles tend to occur in domiciliary and peridomiciliary areas. The presented situation has been observed in Brazil and in other South American countries related to AVL [3]. A recent study in Costa Rica brought to light a very interesting discussion involving environmental changes associated with deforestation, land use, socio-economic condition of human populations and its influence on some sand fly vectors density and diversity. Evidence supports that socio economic inequities and environmental issues could be determinant factors in ACL transmission [4].

The application of ecological niche modeling has been useful and important to predict future scenarios on leishmaniases. In recent analysis using this methodology it was possible to predict the expansion of ACL in North America due to environmental changes, considerably enlarging the number of people at risk of contracting the disease [5].

The Health Department of State of Tocantins already considers that environmental impacts from plant construction may have contributed to the emergence of epidemic outbreaks of leishmaniases in Porto Nacional in 2001 and 2002, when a great number of human cases of AVL were recorded, and also to an epidemic in Palmas, the state capital, in 2003. Over the past three years, Porto Nacional remains endemic for AVL and ACL and has been considered a region of intense transmission of AVL according to Brazilian's Health Ministry classification [2].

The objective of this study is to characterize Phlebotomine fauna in the state of Tocantins and to discuss potential impacts that environmental changes might have on the ecology of leishmaniasis in the municipality of Porto Nacional.

## Materials and Methods

### Ethics Statement

The oral consent to carry out the catches of sand flies was obtained by the Health Department of State of Tocantins during the activities of entomological surveillance.The capture of sand flies by using CDC light traps was carried out in a peridomestic environment, not bringing harm or disturbance of any kind or changing aspects of the inhabitants routines.

### Study area

**Figure 1:**
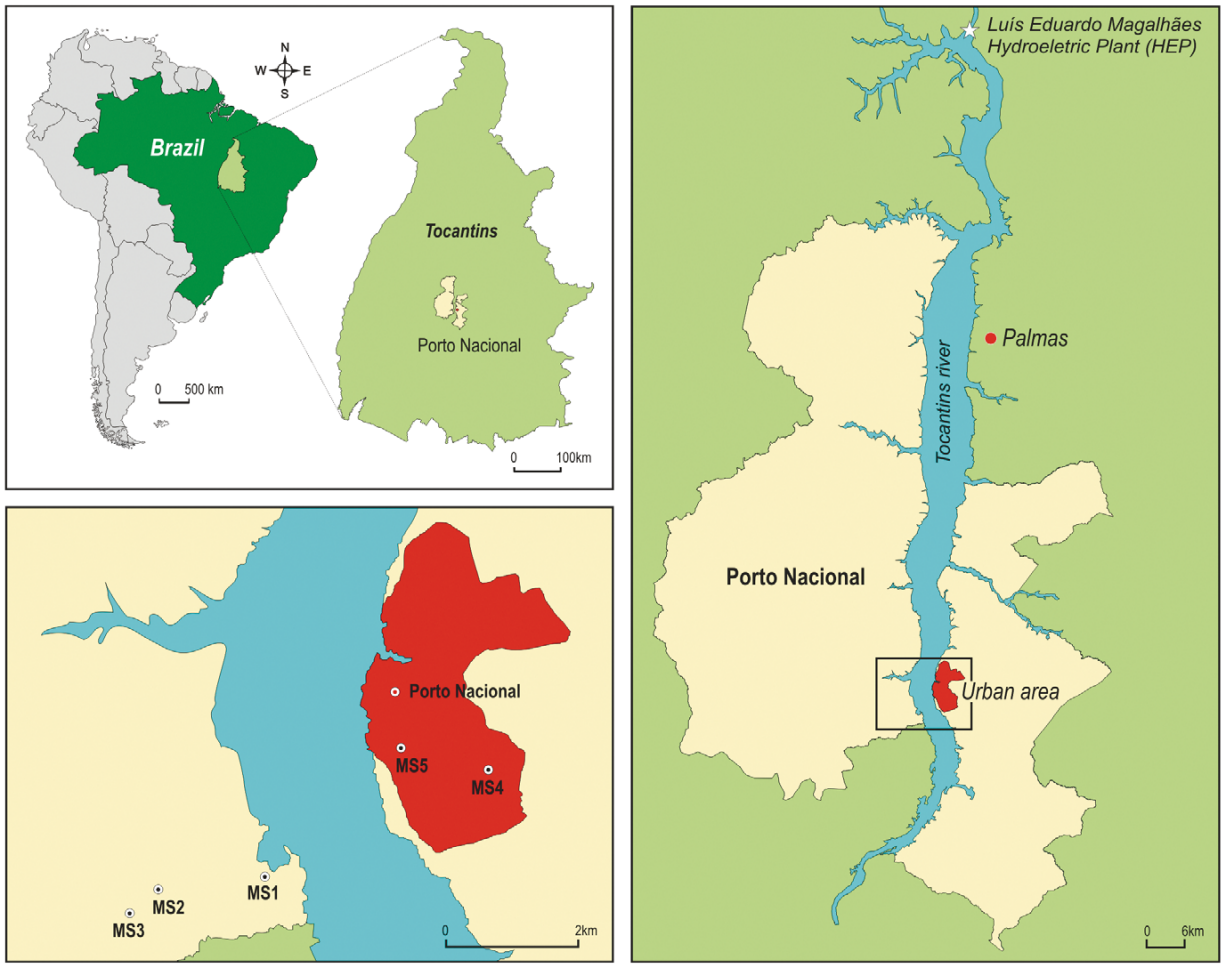
Porto Nacional City, Tocantins State. HEP: Hydroelectric Plant. MS: Sand fly Monitoring Stations.

Porto Nacional is one of the largest cities of the State of Tocantins (Fig. 1). It is geographically situated on coordinates 10°42′29″S and 48°25′02″W. The maximum altitude consists of 212 meters. The climate is classified as tropical and cerrado (Brazilian savannah) is the dominant vegetation [6]. This municipality was directly impacted by the construction of hydroeletric plant Luís Eduardo Magalhães (HEP – Lajeado) on Tocantins river.

Its construction started in July 1998 and finished in November 2001, when the first energy generating unit was triggered, followed by the other four ones in March, May, July and November 2002, respectively. Therefore, a lake of 630 km2 was formed covering the municipalities of Miracema, Lajeado, Palmas, Porto Nacional, Brejinho de Nazareth and Ipueiras. The hydroelectric is located between 9°45′26″S and 48°22′12″W, in the municipality of Lajeado [7].

### Sand fly monitoring station (catch sites)

Sand fly monitoring was carried out in peridomestic sites of rural and urban areas. Five monitoring stations (MS) were chosen: three in rural areas and two in urban areas. Monitoring stations were selected according to epidemiological and socioeconomic conditions of the region. The inclusion criteria considered areas with suitable environments for breeding of the vector (proximity of the forest, presence of pets, shaded and humid shelters) and those with records of human cases of leishmaniases.

Three monitoring stations were established on two rural farms, Gorgulho and Lava Couro: MS1: 10°44′53.7″S, 48°26′41.2″W; MS2: 10°45′2.5″S; 48°28′8.6″W; MS3: 10°45′21.3″S, 48°28′32.7″W. The residences were close to residual forest and had no basic sanitation. Chicken, pigs and dogs were raised in peridomiciliary area.

Two stations were selected in the urban area, Bairro Nacional: MS4: 10°43′31.2″S, 48°23′35.9″W and Bairro Jardim Brasília MS5: 10°43′12.6″S, 48°24′47.4″W (Figure 1).

### Sand flies captures

CDC light traps [8] were installed at the monitoring stations (one trap per peridomicile of each monitoring station) from 17:00 to 07:00. Captures were carried out monthly for three consecutive nights. Meteorological data for the period of captures were obtained from the Instituto Nacional de Meteorologia (INMET, Brazil) station in Porto Nacional.

Sand fly specimens were identified following the taxonomic key by Galati (1995; 2003) [9], [10] and the abbreviation of genus and subgenus names of Marcondes (2007) [11].

### Data analysis

The Index of Species Abundance (ISA) and Standardized Index of Species Abundance (SISA) [12] were used to analyze data obtained from captures in rural and urban areas. Excell 2003 was used for data analysis. Data were recorded in a table and distributed in lines for species and columns for monitoring stations or captures period. Each column was classified separately, according to the number of specimens of each species. The highest value for each column was classified as 1, the second as 2 and so forth. ISA was calculated according to the following formula:
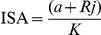
Where:

K: number of columns in the table (number of months collected)
a: number of absences of species per K months multiplied by c
c: the highest position of the species in K columns plus 1
Rj: sum of ratings in each species

The minimum and maximum limits of this index will be determined according to the highest classification of the distribution, so this limit is different in each data series. In order to avoid this variation and standardize the index, that can be converted to a scale of values between 0 and 1 from the calculation of SISA:
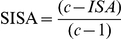



The species is considered the most abundant when its SISA value is closer to the maximum (1). The results provide information about the relative abundance of species as well as about the spatial distribution of collected individuals.

To define the association between the collected sand flies and climate data, univariated correlation analysis (Pearson correlation - *r*) was used. The calculated value of *r* indicates:

- Strong correlation if above 0.7.
- Moderate correlation if between 0.3 and 0.7.
- Weak correlation if between 0.1 and 0.3.
- Insignificant correlation if below 0.1.

To compare the total of sand flies captured in the rural and urban areas, the DivEs software – Diversidade de Espécies v2.0 [13] was used, so that data could be analyzed by the following tests: Shannon-Wiener Diversity Index (H) and Evenness Index (J).

## Results

During 40 months (June 2004 to September 2007) 7162 sand flies were collected, 4533 males and 2629 females, distributed in a total of 48 species detected. The analysis of the abundance index revealed three predominant species: *Lutzomyia longipalpis* (SISA = 1.000), *Nyssomyia whitmani* (SISA = 0.932) and *Sciopemyia sordellii* (SISA = 0.902) (Table S1).

Among the identified species, the following are vectors of leishmaniases: *Lu. longipalpis*, *Migonemyia migonei*, *Brichromomyia. flaviscutellata*, *Nyssomyia antunesi*, *Ny. whitmani* and *Ny. intermedia*.

In rural areas, with over 5400 hours of catches, 2366 sand flies were captured belonging to 45 species. Twenty-two species did not occur in urban areas: *Lutzomyia sherlocki*, *Lu gomezi*, *Sciopemyia microps*, *Evandromyia. sallesi*, *Ev. bacula*, *Ev. walkeri*, *Ev. begonae*, *Ev. pinottii*, *Ev. brachyphala*, *Viannamyia furcata*, *Psathyromyia dasymera*, *Pa. pascalei*, *Pa. brasiliensis*, *Trichopygomyia dasypodogeton*, *Nyssomyia intermedia*, *Ny. richardwardi*, *Martinsmyia oliveirai*, *Mt. minasensis*, *Mi. quinquefer*, *Mi. rorotaensis*, *Pintomyia misionensis* e *Mi. acanthopharynx* (Table S2). The three more abundant species in this environment were respectively: *Lu. longipalpis* (SISA = 1.000), *Sc. sordellii* (SISA = 0.944) e *Ny. whitmani* (SISA = 0.932) (Table S2).

In urban areas, with more than 3600 hours of catches, 4796 sand flies were captured, belonging to 24 species (Table S2), with only three exclusive species for this environment: *Mg. migonei*, *Ev. teratodes* and *Pa. dendrophyla*. The predominant species were respectively: *Lu. longipalpis* (SISA = 1.000), *Pa. punctigeniculata* (SISA = 0.886) and *Ny. whitmani* (SISA = 0.886) (Table S2).

The analysis of species diversity in the studied areas was assessed using the Shannon-Wiener Diversity Index (H) and Evenness Index (J). The rural area (H = 0.8602 and J = 0.5203), compared to urban area (H = 0.3775 and J = 0.27), presented lower abundance of sand flies, although the diversity of species found was higher in rural environment (Table S2).

**Figure 2:**
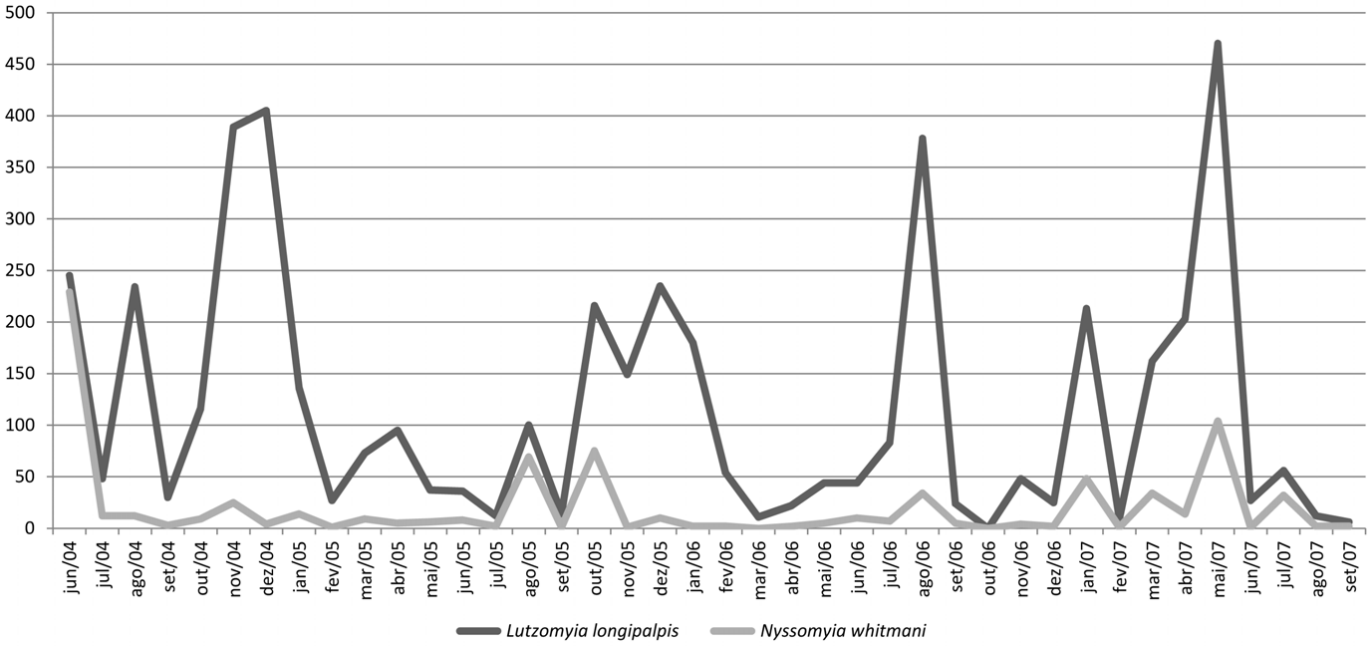
Seasonality of *Lutzomyia longipalpis* and *Nyssomyia whitmani*. Porto Nacional. June 2004–September 2007.

**Figure 3:**
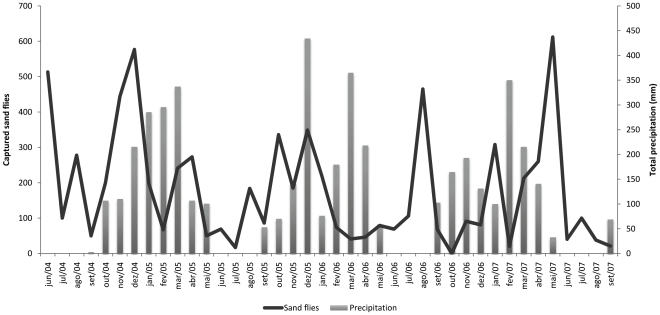
Number of captured sand flies and monthly total precipitation. Porto Nacional. June 2004–September 2007.

**Figure 4:**
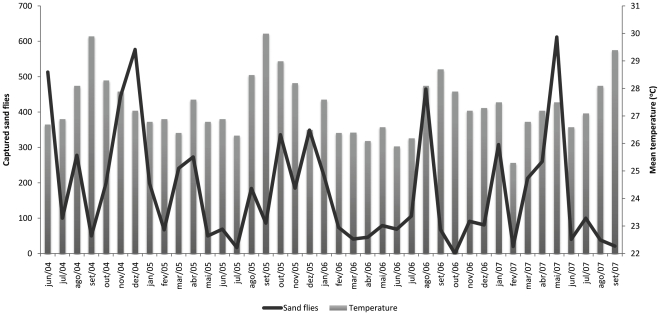
Number of captured sand flies and monthly mean temperature. Porto Nacional. June 2004–September 2007.

**Figure 5:**
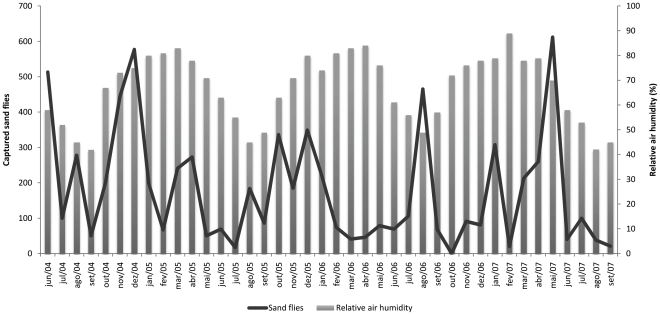
Number of captured sand flies and monthly relative air humidity. Porto Nacional. June 2004–September 2007.

The seasonality of the two most abundant species of medical importance, *Lu. longipalpis* and *Ny. whitmani* is shown in Figure 2. There was no significant correlation between the collected sand flies and climatic data (*r* = −0.006 for precipitation, *r* = 0.091 for temperature, *r* = 0.089 for relative humidity) (Figs. 3, 4 and 5).

## Discussion

As a result of the amount of labor required in the construction of dams in general, migration tends to increase significantly in its surrounding areas. Consequently, a growth in the number of unplanned settlements and deforestation, associated with other environmental impacts, may be observed. Those factors can facilitate the spread of leshmaniases. Another consequence of this context might be the establishment of the enzootic cycle in domiciliary areas, which could mean that these environmental changes cause the expansion of leshmaniases through closer contact between man and the disease's vector.

Both ACL and AVL remain endemic in Porto Nacional since 2001, after HEP Lajeado began operating. In 2010, the Health Department of the State of Tocantins ranked Porto Nacional as one of the three municipalities with the highest frequency of human cases of AVL in the state, from January 2010 to January 2011. Therefore, the city was indicated by the Brazilian Health Ministry as one of the priority areas for disease control actions [14].

Early studies on the phlebotomine fauna from the state of Tocantins have focused on the description of new species [15], [16], [17], [18]; surveys on phlebotomine fauna were carried out in a few municipalities: Alvorada, Itacajá and São Sebastião [19]. Recently, studies carried out in four municipalities (Paraíso do Tocantins, Monte do Carmo, Monte Santo and Porto Nacional) recorded 32 species of sand flies, and among them, some potential vectors of leishmaniasis: *Lu. longipalpis*, *Ny. whitmani*, *Ny. antunesi*, *Bi. flaviscutellata* [3]. In 2004 a new species was described, *Micropygomyia (Silvamyia) echinatopharynx* from the municipality of Porto Nacional [20].

Considering the total of captures, with over 7700 hours, among the 51 identified species, the following were identified for the first time within the state of Tocantins: *Lu. sherlocki*, *Sc. microps*, *Mg. migonei*, *Ev. corumbaensis*, *Ev. begonae*, *Ev. pinottii*, *Ev. brachyphala*, *Vi. furcata*, *Pa. dasymera*, *Pa. lutziana*, *Pa. punctigeniculata*, *Pa. dendrophyla*, *Pa. inflata*, *Pa. pascalei*, *Pa. brasiliensis*, *Ty. dasypodogeton*, *Ny. richardwardi*, *Mt. minasensis*, *Mi. quinquefer*, *Mi. villelai*, *Mi. acanthopharynx*, *Expapillata. cerrandicola*.

The entomologic biodiversity of the Cerrado biome, the second largest vegetation type in Brazil, is not completely known. According to Instituto Brasileiro de Geografia e Estatística (IBGE), Tocantins is one of the states with the greatest degree of deforestation of the Cerrado biome (12,198 km^2^) [21]. The present study brings an important contribution to the knowledge of sand flies, as a group of insects, from Tocantins' biome.

A higher diversity of sand fly species was observed in rural environments, but a greater abundance of sand flies was captured in urban environments.

Similar evidence has been observed previously [6]: a higher diversity of species in the rural area, a higher number of insects collected around houses, and a lower number of species. Studies in a transmission area of ACL revealed lower diversity and abundance of sand fly species inside the domicile than in the peridomiciliary area, due to the high frequency of *Nyssomyia whitmani*, and low density of *Lu. longipalpis* [22]. In Porto Nacional it is observable that in both rural and urban areas *Lu. longipalpis* was the most frequent species.

The diversity of species found in rural areas may be explained by close contact of residences with wild forests, and also by the presence of domestic animal shelters, allowing favorable conditions for the establishment of the cycle of sand flies.

Among such a diverse fauna, we found the following sand fly species identified as vectors of leishmaniases within Porto Nacional: *Lu. longipalpis* and *Mg. migonei*, for AVL, *Mg. migonei*, *Bi. flaviscutellata*; *Ny. antunesi*, *Ny. whitmani* and *Ny. intermedia*, for ACL. Among these, the most abundant species are, respectively, the important vectors of AVL and ACL in Brazil: *Lu. longipalpis* and *Ny. whitmani*.

*Lutzomyia longipalpis* was more frequent in the urban area when compared to rural one. Other studies have already demonstrated that *Lu. longipalpis* easily adapts to the urban environment due to factors not yet clear, but certainly related to environmental conditions for the establishment of breeding places in the disturbed habitat [23]. However, it must be considered that biological factors inherent to the vector, especially the feeding plasticity of adults associated with their high anthropophily, contribute to the installation of the transmission cycle of AVL in urban environment and its maintenance in rural areas at the same time [24]. Clearly, the monitoring stations of this study present environmental characteristics that contribute to enhance the adaptation of the vector to the peridomestic sites, including food sources for adult sand flies and decaying organic matter as breeding sites, common on both studied areas.

Studies have suggested that *Ny. whitmani* is the most important vector of ACL in Brazil, associated with *Leishmania (V.) braziliensis* [25], [26], [27]. This species is present in various brazilian biomes (Amazon Forest, Cerrado, Caatinga and Atlantic Forest) and has adapted to different climatic conditions; it is able to inhabit the intra and peridomicile in impacted areas [19], [20], [21]. It can be inferred that, along with *Lu. longipalpis*, they represent the vectors of leishmaniasis species best adapted to new environmental conditions.

Both *Ny. whitmani* and *Lu. longipalpis* were present in all studied monitoring stations, whether in rural or urban environment. Assuming that *Ny. whitmani* has the capacity to adapt to environmental changes, it is possible that ACL in Porto Nacional has expanded from an enzootic cycle in the first instance to the rural environment and, subsequently, to the urban perimeter (periphery of town), after the construction of HEP Lajeado. Currently, the epidemiology of ACL in Tocantins is clearly associated with occupational behavior, where males represent 76% of registered cases, with 50% of activities related to the agricultural sector [28].

Although expressed in very discrete values, the record of *Bi. flaviscutellata* in periurban area of Porto Nacional draws attention. This sand fly species is the vector of a severe form of ACL associated with *Leishmania (Leishmania) amazonensis*. The possibility that the enzootic cycle of *L. (L.) amazonensis* associated with *Bi. flaviscutellata* could be surviving in secondary forest and that they could even become peridomestic has been previously discussed [29]. This would happen in part, as a consequence of the vector's adaptation to the anthropogenic environment alterations. In fact, in recent years, the dispersal process of this sand fly vector can be observed next to domestic animal shelters, especially in some areas of Cerrado [30], [31]. We can infer that the occurrence of autochthonous human cases by *L. (L.) amazonensis* in the state of Tocantins, would be the result of the dispersion of *Bi. flaviscutellata*, contradicting the hypothesis that leishmaniasis could disappear with the destruction of primary forest [32].

## Supporting Information

Table S1 **Phlebotomine species captured with CDC light traps.** **Porto Nacional. June 2004–September 2007.** Legend: M = male; F = female; % = frequency; SISA = Standardized Index of Species Abundance; FC = final classification according to SISA; * vector species.
(DOC)

Table S2 **Phlebotomine species captured with CDC light traps on the rural and urban area of Porto Nacional.** **June 2004–September 2007.** Legend: M = male; F = female; % = frequency; SISA = Standardized Index of Species Abundance; FC = final classification according to SISA; * vector species.
(DOC)

## References

[1] Brasil. Ministério da Saúde. Secretaria de Vigilância em Saúde (2007). Manual de Vigilância da Leishmaniose Tegumentar, 2^a^ ed.

[2] Brasil. Ministério da Saúde. Secretaria de Vigilância em Saúde (2006). Manual de Vigilância e Controle da Leishmaniose Visceral.

[3] World Health Organization (2010).

[4] Chaves LF, Cohen JM, Pascual M, Wilson ML (2008). Social Exclusion Modifies Climate and Deforestation Impacts on a Vector-Borne Disease.. PLoS Negl Trop Dis.

[5] Gonzalez C, Wang O, Strutz SE, Gonzalez-Salazar C, Sanchez-Cordero V (2010). Climate Change and Risk of Leishmaniasis in North America: Predictions from Ecological Niche Models of Vector and Reservoir Species.. PLoS Negl Trop Dis.

[6] Andrade-Filho JD, Valente MB, de Andrade WA, Brazil RP, Falcão AL (2001). Flebotomíneos do Estado de Tocantins, Brasil (Diptera: Psychodidae).. Rev Soc Bras Med Trop.

[7] Grupo Rede (2003). Usina Hidrelétrica Luís Eduardo Magalhães.. http://www.gruporede.com.br/empresas/princ_lajeado.asp.

[8] Sudia WD, Chamberlain RW (1962). Battery operated light trap, an improved model.. Mosq News.

[9] Galati EAB (1995). Phylogenetic systematics of Phlebotominae (Diptera, Psychodidae) with emphasis on American groups.. Bol Dir Malariol Saneam Amb.

[10] Galati EAB, Rangel EF, Lainson R (2003). Morfologia e Taxonomia.. Flebotomíneos do Brasil.

[11] Marcondes CB (2007). A proposal of generic and subgeneric abbreviations for Phlebotomine sandflies (Diptera: Psychodidae: Phlebotominae) of the world.. Entomol News.

[12] Roberts DR, Hsi BP (1979). An Index of Species Abundance for Use with Mosquito Surveillance Data.. Environ Entomol.

[13] Rodrigues WC (2005). DivEs - Diversidade de espécies. Versão 2.0. Software e Guia do Usuário.. http://www.ebras.bio.br.

[14] Governo do Estado de Tocantins (2010). Informe Entomo-epidemiológico das Leishmanioses.. http://www.saude.to.gov.br.

[15] Barreto MP (1946). Uma nova espécie de flebótomo do Estado de Goiás, Brasil, e chave para determinação das espécies afins (Diptera, Psychodidae).. Rev Bras Biol.

[16] Martins AV, Falcão AL, Silva JE (1962). Nota sobre os flebotomíneos do Estado de Goiás, com a descrição de duas espécies novas e da fêmea de *Lutzomyia longipennis* (Barreto, 1946) e a redescrição do macho de *L. evandroi* (Costa Lima & Antunes, 1936) (Diptera, Psychodidae).. Rev Bras Malariol Doencas Trop.

[17] Martins AV, Falcão AL, Silva JE (1964). Um novo flebótomo do estado de Goiás, *Lutzomyia teratodes sp. n.* (Diptera, Psychodidae).. Rev Bras Biol.

[18] Martins AV, Falcão AL, Silva JE (1975). Descrição de fêmea de *Lutzomyia teratodes* Martins, Falcão & Silva 1964 (Diptera, Psychodidae, Phlebotominae).. Rev Bras Biol.

[19] Lustosa ED, Naves HP, Carvalho MESD, Barbosa W (1986). Contribuição ao conhecimento da fauna flebotomínica do Estado de Goiás – 1984–1985.. Nota Prévia I Rev Pat Trop.

[20] Andrade-Filho JD, Galati EA, de Andrade WA, Falcão AL (2004). Description of *Micropygomyia (Silvamyia) echinatopharynx sp. nov.* (Diptera: Psychodidae) a new species of phlebotominae sand fly from the state of Tocantins, Brazil.. Mem Inst Oswaldo Cruz.

[21] IBGE. Instituto Brasileiro de Geografia e Estatística (2010). http://www.ibge.gov.br.

[22] Loiola CF, Silva DA, Galati EAB (2007). Phlebotomine fauna (Diptera: Psychodidae) and species abundance in an endemic area of American cutaneous leishmaniasis in southeastern Minas Gerais, Brazil.. Mem Inst Oswaldo Cruz.

[23] Maia-Elkhoury AN, Alves WA, Sousa-Gomes ML, Sena JM, Luna EA (2008). Visceral leishmaniasis in Brazil: trends and challenges.. Cad Saude Publica.

[24] Rangel EF, Vilela ML (2008). *Lutzomyia longipalpis* (Diptera, Psychodidae, Phlebotominae) and urbanization of visceral leishmaniasis in Brazil.. Cad Saude Publica.

[25] Costa SM, Cechinel M, Bandeira V, Zannuncio JC, Lainson R (2007). *Lutzomyia (Nyssomyia) whitmani s.l.* (Antunes & Coutinho, 1939) (Diptera: Psychodidae: Phlebotominae): geographical distribution and the epidemiology of American cutaneous leishmaniasis in Brazil – Mini-review.. Mem Inst Oswaldo Cruz.

[26] Rangel EF (2010). *Lutzomyia (Nyssomyia) whitmani* and the eco-epidemiology of American Cutaneous Leishmaniasis in Brazil.. Workshop de Genética e Biologia Molecular de Insetos Vetores de Doenças Tropicais.

[27] Rangel EF, Lainson R (2009). Proven and putative vectors of American cutaneous leishmaniasis in Brazil: aspects of their biology and vectorial competence.. Mem Inst Oswaldo Cruz.

[28] Bigeli JG, Azevedo CG, Pereira MGA, Costa JNG (2010). Perfil da Leishmaniose Tegumentar Americana no Estado de Tocantins.. Anais XIV Reunião de Pesquisa Aplicada em Leishmanioses. Uberaba.

[29] Lainson R, Shaw JJ, Silveira FT, de Souza AA, Braga RR (1994). The dermal leishmaniases of Brazil, with special reference to the eco-epidemiology of the disease in Amazônia.. Mem Inst Oswaldo Cruz.

[30] Lainson R, Rangel EF (2009). Proven and putative vectors of American cutaneous leishmaniasis in Brazil: aspects of their biology and vectorial competence.. Mem Inst Oswaldo Cruz.

[31] Vilela ML, Azevedo ACR, Costa SM, Costa WA, Motta-Silva D (2008). Sand fly survey in the influence area of Peixe Angical hydroelectric plant, State of Tocantins, Brazil..

[32] Campbell-Lendrum D, Dujardin J, Martinez E, Feliciangeli MD, Perez JE (2001). Domestic and Peridomestic Transmission of American Cutaneous Leishmaniasis: Changing Epidemiological Patterns Present New Control Opportunities.. Mem Inst Oswaldo Cruz.

