# Supplementary material for: Phlebotomine Fauna (Diptera: Psychodidae) and Putative Vectors of Leishmaniases in Impacted Area by Hydroelectric Plant, State of Tocantins, Brazil
Source: PLoS One. 2011 Dec 7;6(12):e27721. doi: 10.1371/journal.pone.0027721 (PMC3233550; doi:10.1371/journal.pone.0027721)
Supplement: Table S1 — Phlebotomine species captured with CDC light traps. Porto Nacional. June 2004–September 2007. Legend: M = male; F = female; % = frequency; SISA = Standardized Index of Species Abundance; FC = final classification according to SISA; * vector species. (DOC) [file pone.0027721.s001.doc]

| **Species** | **M** | **F** | **Total** | **%** | **SISA** | **FC** |
| --- | --- | --- | --- | --- | --- | --- |
| *Brumptomyia brumpti* | 8 | 8 | 16 | 0,22 | 0,275 | 16º |
| *Br.* spp. | 1 | 1 | 2 | 0,03 | - | - |
| *Micropygomyia acanthopharynx* | 0 | 3 | 3 | 0,04 | 0,11 | 33º |
| *Mi. peresi* | 2 | 1 | 3 | 0,04 | 0,166 | 25º |
| *Mi. quinquefer* | 6 | 7 | 13 | 0,18 | 0,298 | 14º |
| *Mi. rorotaensis* | 3 | 2 | 5 | 0,07 | 0,139 | 28º |
| *Mi. villelai* | 17 | 60 | 77 | 1,08 | 0,773 | 6º |
| *Mi. micropyga* | 6 | 2 | 8 | 0,11 | 0,278 | 15º |
| *Mi.* spp. | 0 | 2 | 2 | 0,03 | - | - |
| *Sciopemyia microps* | 3 | 1 | 4 | 0,06 | 0,129 | 30º |
| *Sc. sordellii* | 123 | 322 | 445 | 6,21 | 0,902 | 3º |
| *Lutzomyia gomezi** | 0 | 2 | 2 | 0,03 | 0,102 | 34º |
| *Lu sherlocki* | 0 | 6 | 6 | 0,08 | 0,227 | 19º |
| *Lu. longipalpis** | 3491 | 1191 | 4682 | 65,37 | 1,000 | 1º |
| *Lu.* spp. | 25 | 47 | 72 | 1,01 | - | - |
| *Migonemyia migonei** | 1 | 0 | 1 | 0,01 | 0,088 | 35º |
| *Mg.* sp. | 1 | 0 | 1 | 0,01 | - | - |
| *Pintomyia misionensis* | 0 | 1 | 1 | 0,01 | 0,01 | 48º |
| *Expapillata cerrandicola* | 0 | 1 | 1 | 0,01 | 0,07 | 38º |
| *Ex.* sp. | 0 | 1 | 1 | 0,01 | - | - |
| *Trichopygomyia dasypodogeton* | 3 | 2 | 5 | 0,07 | 0,197 | 23º |
| *Evandromyia bacula* | 1 | 0 | 1 | 0,01 | 0,007 | 44º |
| *Ev. carmelinoi* | 186 | 129 | 315 | 4,40 | 0,885 | 4º |
| *Ev. evandroi* | 20 | 32 | 52 | 0,73 | 0,746 | 8º |
| *Ev. lenti* | 39 | 27 | 66 | 0,92 | 0,702 | 10º |
| *Ev. termitophila* | 16 | 67 | 83 | 1,16 | 0,759 | 7º |
| *Ev. walkeri* | 1 | 2 | 3 | 0,04 | 0,132 | 29º |
| *Ev. begonae* | 0 | 1 | 1 | 0,01 | 0,071 | 37º |
| *Ev. brachyphala* | 0 | 1 | 1 | 0,01 | 0,047 | 40º |
| *Ev. pinottii* | 0 | 6 | 6 | 0,08 | 0,149 | 27º |
| *Ev. saulensis* | 1 | 5 | 6 | 0,08 | 0,241 | 17º |
| *Ev. teratodes* | 0 | 1 | 1 | 0,01 | 0,088 | 36º |
| *Ev. corumbaensis* | 0 | 2 | 2 | 0,03 | 0,112 | 31º |
| *Ev. sallesi* | 1 | 0 | 1 | 0,01 | 0,047 | 39º |
| *Psathyromyia aragaoi* | 14 | 4 | 18 | 0,25 | 0,471 | 12º |
| *Pa. brasiliensis* | 0 | 1 | 1 | 0,01 | 0,007 | 46º |
| *Pa. inflata* | 0 | 3 | 3 | 0,04 | 0,200 | 22º |
| *Pa. lutziana* | 1 | 2 | 3 | 0,04 | 0,190 | 24º |
| *Pa. pascalei* | 0 | 3 | 3 | 0,04 | 0,108 | 32º |
| *Pa. hermanlenti* | 4 | 12 | 16 | 0,22 | 0,580 | 11º |
| *Pa. campbelli* | 1 | 6 | 7 | 0,10 | 0,163 | 26º |
| *Pa. dasymera* | 0 | 1 | 1 | 0,01 | 0,047 | 41º |
| *Pa. dendrophyla* | 0 | 3 | 3 | 0,04 | 0,217 | 21º |
| *Pa. punctigeniculata* | 16 | 197 | 213 | 2,97 | 0,742 | 9º |
| *Pa. shannoni* | 2 | 2 | 4 | 0,06 | 0,234 | 18º |
| *Pa.* sp. | 0 | 1 | 1 | 0,01 | - | - |
| *Viannamyia furcata* | 0 | 1 | 1 | 0,01 | 0,007 | 45º |
| *Martinsmyia minasensis* | 1 | 0 | 1 | 0,01 | 0,007 | 47º |
| *Mt. oliveirai* | 7 | 11 | 18 | 0,25 | 0,319 | 13º |
| *Bichoromyia flaviscutellata** | 0 | 7 | 7 | 0,10 | 0,220 | 20º |
| *Psychodopygus* spp. | 1 | 2 | 3 | 0,04 | - | - |
| *Nyssomyia antunesi** | 60 | 107 | 167 | 2,33 | 0,803 | 5º |
| *Ny. intermedia** | 0 | 1 | 1 | 0,01 | 0,047 | 42º |
| *Ny. richardwardi* | 1 | 0 | 1 | 0,01 | 0,047 | 43º |
| *Ny. whitmani** | 470 | 332 | 802 | 11,20 | 0,932 | 2º |
| Total | 4533 | 2629 | 7162 | 100 | - | - |
