# Supplementary material for: Phlebotomine Fauna (Diptera: Psychodidae) and Putative Vectors of Leishmaniases in Impacted Area by Hydroelectric Plant, State of Tocantins, Brazil
Source: PLoS One. 2011 Dec 7;6(12):e27721. doi: 10.1371/journal.pone.0027721 (PMC3233550; doi:10.1371/journal.pone.0027721)
Supplement: Table S2 — Phlebotomine species captured with CDC light traps on the rural and urban area of Porto Nacional. June 2004–September 2007. Legend: M = male; F = female; % = frequency; SISA = Standardized Index of Species Abundance; FC = final classification according to SISA; * vector species. (DOC) [file pone.0027721.s002.doc]

| **Species** | **Rural Area** | | | | | | **Urban Area** | | | | | |
| --- | --- | --- | --- | --- | --- | --- | --- | --- | --- | --- | --- | --- |
|  | **M** | **F** | **Total** | **%** | **SISA** | **CF** | **M** | **F** | **Total** | **%** | **SISA** | **FC** |
| *Brumptomyia brumpti* | 8 | 8 | 16 | 0,68 | 0,458 | 15º | 0 | 0 | 0 | - | - | - |
| *Br.* spp. | 1 | 0 | 1 | 0,04 | - | - | 0 | 1 | 1 | - | - | - |
| *Micropygomyia acanthopharynx* | 0 | 3 | 3 | 0,13 | 0,181 | 25º | 0 | 0 | 0 | - | - | - |
| *Mi. peresi* | 2 | 0 | 2 | 0,08 | 0,130 | 29º | 0 | 1 | 1 | 0,02 | 0,029 | 24º |
| *Mi. quinquefer* | 6 | 7 | 13 | 0,55 | 0,497 | 14º | 0 | 0 | 0 | - | - | - |
| *Mi. rorotaensis* | 3 | 2 | 5 | 0,21 | 0,232 | 20º | 0 | 0 | 0 | - | - | - |
| *Mi. villelai* | 9 | 32 | 41 | 1,69 | 0,780 | 6º | 8 | 28 | 36 | 0,75 | 0,600 | 7º |
| *Mi. micropyga* | 5 | 1 | 6 | 0,25 | 0,260 | 18º | 1 | 1 | 2 | 0,04 | 0,171 | 17º |
| *Mi.* spp. | 0 | 2 | 2 | 0,08 | - | - | 0 | 0 | 0 | - | - | - |
| *Sciopemyia microps* | 3 | 1 | 4 | 0,17 | 0,215 | 23º | 0 | 0 | 0 | - | - | - |
| *Sc. sordellii* | 114 | 288 | 402 | 17,03 | 0,944 | 2º | 9 | 34 | 43 | 0,9 | 0,729 | 5º |
| *Lutzomyia gomezi* | 0 | 2 | 2 | 0,08 | 0,169 | 26º | 0 | 0 | 0 | - | - | - |
| *Lu. sherlocki* | 0 | 6 | 6 | 0,08 | 0,379 | 16º | 0 | 0 | 0 | - | - | - |
| *Lu. longipalpis** | 413 | 397 | 810 | 35,42 | 1,000 | 1º | 3078 | 794 | 3872 | 80,7 | 1,000 | 1º |
| *Lu.* spp. | 11 | 15 | 26 | 1,10 | - | - | 0 | 0 | 0 | - | - | - |
| *Migonemyia migonei** | 0 | 0 | 0 | - | - | - | 1 | 0 | 1 | 0,02 | 0,029 | 20º |
| *Mg.* sp | 0 | 0 | 0 | - | - | - | 1 | 0 | 1 | - | - | - |
| *Pintomyia misionensis* | 0 | 1 | 1 | 0,04 | 0,011 | 45º | 0 | 0 | 0 | - | - | - |
| *Expapillata cerrandicola* | 0 | 1 | 1 | 0,04 | 0,119 | 32º | 0 | 0 | 0 | - | - | - |
| *Ex.* sp | 0 | 0 | 0 | - | - | - | 0 | 1 | 1 | - | - | - |
| *Trichopygomyia dasypodogeton* | 3 | 2 | 5 | 0,21 | 0,328 | 17º | 0 | 0 | 0 | - | - | - |
| *Evandromyia bacula* | 1 | 0 | 1 | 0,04 | 0,011 | 39º | 0 | 0 | 0 | - | - | - |
| *Ev. carmelinoi* | 95 | 75 | 168 | 7,06 | 0,887 | 4º | 93 | 54 | 147 | 3,07 | 0,800 | 4º |
| *Ev. evandroi* | 11 | 13 | 24 | 1,31 | 0,751 | 8º | 9 | 19 | 28 | 0,58 | 0,557 | 9º |
| *Ev. lenti* | 23 | 10 | 33 | 1,39 | 0,706 | 9º | 16 | 17 | 33 | 0,69 | 0,486 | 10º |
| *Ev. termitophila* | 14 | 53 | 67 | 2,79 | 0,763 | 7º | 2 | 14 | 16 | 0,33 | 0,586 | 8º |
| *Ev. walkeri* | 1 | 2 | 3 | 0,13 | 0,220 | 22º | 0 | 0 | 0 | - | - | - |
| *Ev. begonae* | 0 | 1 | 1 | 0,04 | 0,119 | 30º | 0 | 0 | 0 | - | - | - |
| *Ev. brachyphala* | 0 | 1 | 1 | 0,04 | 0,079 | 34º | 0 | 0 | 0 | - | - | - |
| *Ev. pinottii* | 0 | 6 | 6 | 0,08 | 0,249 | 19º | 0 | 0 | 0 | - | - | - |
| *Ev. saulensis* | 1 | 4 | 5 | 0,21 | 0,226 | 21º | 0 | 1 | 1 | 0,02 | 0,100 | 19º |
| *Ev. teratodes* | 0 | 0 | 0 | - | - | - | 0 | 1 | 1 | 0,02 | 0,029 | 21º |
| *Ev. corumbaensis* | 0 | 1 | 1 | 0,04 | 0,011 | 38º | 0 | 1 | 1 | 0,02 | 0,100 | 18º |
| *Ev. sallesi* | 1 | 0 | 1 | 0,04 | 0,079 | 33º | 0 | 0 | 0 | - | - | - |
| *Psathyromyia aragaoi* | 13 | 4 | 17 | 0,72 | 0,638 | 10º | 1 | 0 | 1 | 0,02 | 0,029 | 23º |
| *Pa. brasiliensis* | 0 | 1 | 1 | 0,04 | 0,011 | 43º | 0 | 0 | 0 | - | - | - |
| *Pa. inflata* | 0 | 1 | 1 | 0,04 | 0,119 | 31º | 0 | 2 | 2 | 0,04 | 0,200 | 16º |
| *Pa. lutziana* | 0 | 2 | 2 | 0,08 | 0,169 | 27º | 1 | 0 | 1 | 0,02 | 0,029 | 22º |
| *Pa. pascalei* | 1 | 2 | 3 | 0,13 | 0,181 | 24º | 0 | 0 | 0 | - | - | - |
| *Pa. hermanlenti* | 4 | 9 | 13 | 0,55 | 0,588 | 12º | 0 | 3 | 3 | 0,06 | 0,271 | 13º |
| *Pa. campbelli* | 1 | 0 | 1 | 0,04 | 0,011 | 41º | 0 | 6 | 6 | 0,13 | 0,314 | 11º |
| *Pa. dasymera* | 0 | 1 | 1 | 0,04 | 0,079 | 35º | 0 | 0 | 0 | - | - | - |
| *Pa. dendrophyla* | 0 | 0 | 0 | - | - | - | 0 | 3 | 3 | 0,06 | 0,229 | 15º |
| *Pa. punctigeniculata* | 3 | 10 | 13 | 0,55 | 0,616 | 11º | 13 | 187 | 200 | 4,17 | 0,886 | 2º |
| *Pa. shannoni* | 0 | 1 | 1 | 0,04 | 0,011 | 42º | 2 | 1 | 3 | 0,06 | 0,271 | 12º |
| *Pa.* sp | 0 | 0 | 0 | - | - | - | 0 | 1 | 1 | - | - | - |
| *Viannamyia furcata* | 0 | 1 | 1 | 0,04 | 0,011 | 40º | 0 | 0 | 0 | - | - | - |
| *Martinsmyia minasensis* | 1 | 0 | 1 | 0,04 | 0,011 | 44º | 0 | 0 | 0 | - | - | - |
| *Mt. oliveirai* | 7 | 11 | 18 | 0,80 | 0,531 | 13º | 0 | 0 | 0 | - | - | - |
| *Bichromomyia flaviscutellata** | 0 | 3 | 3 | 0,13 | 0,136 | 28º | 0 | 4 | 4 | 0,08 | 0,243 | 14º |
| *Psychodopygus* spp. | 1 | 2 | 3 | 0,13 | - | - | 0 | 0 | 0 | - | - | - |
| *Nyssomyia antunesi** | 46 | 51 | 97 | 4,06 | 0,825 | 5º | 14 | 56 | 70 | 1,46 | 0,614 | 6º |
| *Ny. intermedia** | 0 | 1 | 1 | 0,04 | 0,079 | 36º | 0 | 0 | 0 | - | - | - |
| *Ny. richardwardi* | 1 | 0 | 1 | 0,04 | 0,079 | 37º | 0 | 0 | 0 | - | - | - |
| *Ny. whitmani** | 297 | 234 | 531 | 22,61 | 0,932 | 3º | 173 | 98 | 271 | 5,65 | 0,886 | 3º |
| Total | 1097 | 1269 | 2366 | - | - | - | 3436 | 1360 | 4796 | - | - | - |
| Shannon-Wiener Diversity Index (H) | 0,86 | | | | | | 0,38 | | | | | |
| Evenness Index (J) | 0,52 | | | | | | 0,27 | | | | | |
